# Identifying Occupational Therapy Research Priorities in Trinidad and Tobago: A Group Concept Mapping Study

**DOI:** 10.1155/2021/9970566

**Published:** 2021-10-12

**Authors:** Savannah Murray-Mendes, Anastasia Raquel Martinez, Katie L. Hackett

**Affiliations:** ^1^Department of Social Work Education and Community Wellbeing, Northumbria University, Newcastle upon Tyne, UK; ^2^Department of Occupational Therapy, Department of Occupational Therapy, Maracas/St. Joseph, Trinidad and Tobago

## Abstract

In Trinidad and Tobago, occupational therapy is an emerging profession with limitations in the number of practitioners and the scope of practice. With the development of a new Master of Science Occupational Therapy programme in the country, the profession is continuously growing. There has been an increased demand for culturally relevant research to build the occupational therapy evidence base locally. However, the narrow range of occupational therapy literature in the country makes it difficult to highlight research gaps and decipher what research areas should be prioritised to best impact occupational therapy practice at present. This group concept mapping study is aimed at identifying priority areas for occupational therapy research in Trinidad and Tobago from the perspectives of occupational therapy students and practitioners. Participants brainstormed and contributed specific research ideas they would like to see developed in the country. Individually, participants sorted these ideas into themes and rated each idea based on perceived importance and feasibility. Using the GroupWisdom™ software, multidimensional scaling and hierarchical cluster analyses were applied to the sort data to create idea clusters within a concept map. Rating values were analysed to determine priority research themes within the concept map. The resulting concept map illustrated seven research priorities: Contextualising Practitioner Development, The Realities of Emerging OT Practice, Localising Mental Health OT, Occupation and Participation of Children and Youth, School-based OT in the Local Context, OT with Special Populations, and OT Contributions to the Public Health Sector. These findings represent the research needs of the occupational therapy profession in Trinidad and Tobago and will help to focus future researchers' efforts to expand the local evidence base.

## 1. Introduction

In Trinidad and Tobago, occupational therapy is an emerging field with only 22 of 24 registered occupational therapists reported to be practicing in the country as of 2019 [1]. Recent reports by the Ministry of Health (MoH) [2] and the National Policy for Persons with Disabilities (NPPD) focus on the country's need for rehabilitative services, which are essential for primary health care, prevention of noncommunicable diseases, and individuals' independent participation and inclusion in society; this encompasses occupational therapy services [3]. The unanimity of private sector employers, government ministries, public regional health authorities, and policy makers regarding the need for occupational therapists is apparent [4]. In 2016, it was approximated that there was a need for 250 occupational therapists [4]. To combat this growing need, in September 2016, a Master of Science in occupational therapy was launched at the University of the Southern Caribbean in Trinidad. This is the only programme of its nature in the Caribbean region, and it is aimed at alleviating the shortage of occupational therapists that currently exists [4]. By the end of 2020, it was anticipated that this programme would increase the number of professionals in Trinidad and Tobago by 93% [5]. With the increasing growth of occupational therapy in the country, it is crucial that relevant research echoes this advancement, both to ensure the profession remains informed by evidence, and that interventions are appropriate to both the population and within the cultural context.

According to Trinidad and Tobago's occupational therapy Code of Ethics, therapists are responsible for the development of practice through research activity [6]. The MoH [2] and the NPPD [3] reflect the government's commitment to engage in essential research, which ensures that rehabilitative care is based on scientific evidence that considers cultural context and national experience. Iwama [7] argues that occupational therapy, based on current theories and models, is ethnocentric, revolving around Western and European worldviews and sociocultural contexts. He suggests that culturally relevant conceptual models, theories, and modes of practice must be developed for occupational therapy to be truly meaningful to its users. At present, there are only a few published studies that have focused on occupational therapy in the country [4, 5, 8–14]. Many of these studies, however, do not directly contribute to Trinidad and Tobago's occupational therapy literature as they are focused on the experiences of international stakeholders.

Due to the restricted existing occupational therapy literature, it is difficult to determine what research needs should take precedence. Comparable to occupational therapy development in Africa, there is a need for “a strategic plan for practical, appropriate research, which will allow the profession to develop in a cost-effective and efficient manner so that occupational therapy services can reach as much of the population as possible” [15, p. 143]. Research is crucial in expanding the spread of knowledge and developing the profession in an evidence-based manner [16]. By identifying research priorities using systematic strategies, research on specific and important areas is encouraged. When clinicians' perspectives are used to set prioritised areas of research, the results are clinically relevant and can help with creating focus, guidance, and direction for future development of the field [17].

Research priorities for occupational therapy have been explored and identified in a few other countries, such as the United Kingdom [18–21], Ireland [22], and the United States of America [23]. Research priorities have also been developed for specific occupational therapy client groups such as mental health [17, 24–26] and cerebral palsy [27]. The World Federation of Occupational Therapy (WFOT) also administered an international Delphi study where several WFOT member organisations and approved educational programmes identified occupational therapy research priorities [28]. These international research priorities are workable for countries where occupational therapy is relatively prominent but do not adequately represent the needs of occupational therapy in Trinidad and Tobago, since the profession is within the earlier stages of building awareness and gaining traction. This research priority setting study is the first of its kind within the Caribbean context and within Trinidad and Tobago.

The literature therefore shows the undeniable need for more occupational therapy practitioners, services, and research in Trinidad and Tobago [1–5]. With the development of the local Master's programme, the field is expected to grow, and there is a call for culturally relevant research to expand the evidence base [2, 7, 29]. Previous research priority setting studies were successful in targeting important areas for future research, and clinicians' perspectives are useful in creating focus and direction [17]. Therefore, this study is aimed at developing occupational therapy research priorities from the perspectives of local clinicians and students. It is hoped that the findings will encourage development of the field and form a useful decision-making tool for future occupational therapy researchers and possibly other allied health professionals.

## 2. Methods

### 2.1. Study Design

This study used a group concept mapping (GCM), mixed methods, participatory approach to identify research priorities for occupational therapy in Trinidad and Tobago. GCM, developed by Trochim [30], incorporates qualitative individual and group processes (brainstorming, sorting, rating) with quantitative multivariate statistical analyses (multidimensional scaling and hierarchical cluster analysis). GCM is a structured process that allows a group of individuals to express their unique ideas on a topic and gives representation to the relationships between these ideas using visual, two-dimensional concept maps [31]. This method is supported by the online platform GroupWisdom™ (https://groupwisdom.com/) which is specifically designed for GCM projects and allows for the collection, management, and analysis of data. A licence was purchased specifically for this project.

GCM has previously been used for strategic planning, developing research agendas, and addressing healthcare issues [32–34]. Notably, previous research priority-setting studies have mainly used methods such as the Delphi technique [17, 22, 26–28]. However, GCM is the proposed method for this study since it has “strong internal representational validity and very strong sorting and rating reliability” [32, p. 1]. It does not force participants to form a consensus but rather gives participants the opportunity to contribute ideas without validation from others and allows a diverse range of ideas to be represented [30, 35]. This is important because it allows all participants' voices to be heard. Rising et al. [36] found GCM to be an accessible and powerful tool for identifying person-centred priorities for future research. GCM involves 5 specific stages: (1) brainstorming, (2) statement synthesis, (3) sorting, (4) rating, and (5) data analysis [31, 37].

### 2.2. Participants

Purposive sampling was used to identify participants through the Trinidad and Tobago Occupational Therapy Association (TTOTA) whose members consist of occupational therapy practitioners and students. Students were included in this study as they are the future of the occupational profession in the country. A TTOTA member acted as a gatekeeper for this study and assisted with participant recruitment. The same TTOTA member was an active member of the research team and also assisted with the statement synthesis and analysis and stages of the study. Email invitations were sent to all TTOTA members (*n* = 38) with a participant information sheet and a link to an online GroupWisdom™ survey. Participants were asked to engage with the software twice: first to brainstorm ideas and on a second occasion to sort and rate the ideas generated resulting from the brainstorming activity. Participants could take part in the sorting and rating activities even if they had not engaged in the first brainstorming activity [31]. To gain a deeper understanding of the participants' perspectives, each participant was asked to answer demographic questions when they logged into the project for the first time. The demographic questions asked participants to identify: whether they were a student or registered occupational therapist, the number of years practiced in Trinidad and Tobago, their area(s) of current or intended practice, and whether they had experience with occupational therapy research in Trinidad and Tobago.

### 2.3. Ethical Approval

Ethical approval was granted by the Northumbria University Ethics Committee, and online informed consent was obtained from each participant prior to data collection.

### 2.4. Data Collection

#### 2.4.1. Stage 1: Brainstorming

Participants were first invited to anonymously respond to the following focal prompt: “Specific occupational therapy research areas that I would like to see developed in Trinidad and Tobago include…”. They were encouraged to generate as many research ideas as they liked. While participants engaged in this online activity, they could see the anonymised statements generated by other participants who had already taken part. This activity yielded a list of statements from all participants who contributed. The brainstorming activity was closed once all invited TTOTA members had the chance to take part.

#### 2.4.2. Stage 2: Statement Synthesis

The raw list of statements was imported to a spreadsheet, inspected, and edited to produce a final statement set. Statements that expressed more than one idea were split such that each statement presented a single idea. Statements were then assigned a keyword to show their meaning. Statements with similar keywords were examined together, and duplicate ideas were removed. Each statement was then considered for syntax and legibility to produce a final list of refined, unique statements [31, 38].

#### 2.4.3. Stage 3: Sorting Activity

Participants were invited by email a second time to participate in the sorting and rating activities. Each of the final statements was numbered and listed in a random order within the GroupWisdom™ software. Each participant was asked to sort statements into groups according to similarity in meaning, by dragging and dropping in the software, and then give each group a relevant name. Participants needed to have sorted more than 75% of the statements correctly into meaningful themes to be approved for further analysis [31].

#### 2.4.4. Stage 4: Rating Activity

Participants were asked to rate each statement on two 5-point Likert scales for importance and feasibility. The importance scale ranged from 1 (relatively unimportant), 2 (somewhat important), 3 (moderately important), 4 (very important) to 5 (extremely important). The feasibility scale ranged from 1 (not feasible at all), 2 (unfeasible), 3 (neither feasible nor unfeasible), 4 (feasible), to 5 (extremely feasible). Participants were asked to rate each statement relative to the others, so the full range of the scale was utilised. These rating questions, which have been used in other GCM studies [39], were chosen to ascertain the significance of each research idea (importance) and the practicability of researching it within the local context (feasibility). Both sorting and rating activities were closed once all invited TTOTA members had the opportunity to take part.

### 2.5. Analysis

Qualitative and quantitative techniques were used to analyse the resultant data. Firstly, the GroupWisdom™ software aggregated the sort data into a similarity matrix which shows the position of each statement in relation to how it was sorted with others as a point on an *X*-*Y* axis. Through the process of multidimensional scaling, the similarity matrix was used to produce a two-dimensional point map, with each statement visually represented as a point on the map [31]. Points that were located closer together depicted statements that were regularly sorted together since participants thought these statements were conceptually similar [40]. A stress value was generated, which indicated the degree to which the distances on the point map are representative of the sorting data, with lower stress values suggesting more stability and goodness of fit. Stress values below 0.365 are desirable for GCM studies [31].

Secondly, hierarchical cluster analysis was applied to the data using Ward's method [41] to group similar meaning statements into clusters representing how participants arranged the research ideas they generated [42]. This is a statistical technique used within the GroupWisdom™ platform, which involves drawing boundaries around points on the point map to form the cluster map. Clusters are combined one by one such that several varying cluster strategies are viable, and a final cluster map solution must be chosen through qualitative interpretive analysis [42, 43]. In this study, the first author consulted with the TTOTA gatekeeper to examine as many as 13 clusters and as little as 5 to determine whether the statements within each cluster represented the overall theme. Clusters that were located close together and contained similar meaning statements were combined into one. This process was repeated until only distinct clusters remained, and the statements within each cluster were directly linked to the cluster's theme. In any instance where a point was a qualitative outlier (i.e., the statement represented by the point seemed to better fit within the theme of an adjacent cluster), the cluster boundary was redrawn and that point was included in a more suitable cluster [42, 44].

Cluster label analysis was conducted collaboratively with the TTOTA gatekeeper to determine appropriate names for each cluster. The software suggested cluster labels for the final cluster solution based on the names given by the participants during the sorting stage. Cluster label suggestions were used; however, if these were not accurately representative of the statements within the cluster, the original cluster labels were decided upon following discussion with the research team. Engaging in this process with a member of the TTOTA meant that the results would incorporate a local perspective and experience and be more representative of the needs of the local population.

Rated data were then organised into a pattern match which visually represented and enabled the comparison of the mean importance and mean feasibility for each themed cluster [31]. This helped to highlight priority research themes. Individual go-zone graphs were generated for each cluster to indicate which statements within the clusters were the most important and feasible. Go-zones are bivariate graphs that are partitioned into four quadrants by the mean scores for importance and feasibility [31]. The upper-right quadrant, called to “go-zone,” contained statements that were rated above the mean for both importance and feasibility. Statements that were in the “go-zone” were considered to indicate priority research statements. An additional table was generated with a list of all statements arranged by cluster and rating scores. These visual representations of the data are aimed at aiding the appraisal and utilisation of the results for researchers who are interested in different areas of occupational therapy practice.

### 2.6. Rigor and Validity

The collaborative and participatory approach involved in this study promoted the credibility and applicability of the results by ensuring the findings are direct representations of local experiences and perspectives. Collaboration with the TTOTA gatekeeper in the cluster analysis further promoted trustworthiness and rigor.

## 3. Results

### 3.1. Study Population

**Figure 1:**
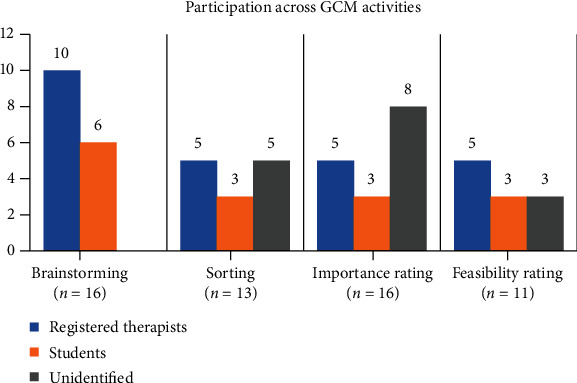
Bar chart showing participation across each GCM activity. Participants were asked to identify as a student or registered occupational therapist. Not all participants responded to demographic questions, leaving some participants' titles as “unidentified.”

Of the approximated 38 TTOTA members invited to participate in this study, 25 took part in one or more of the GCM activities.  Figure 1 shows a breakdown of how many participants took part in each activity and whether they identified themselves as a student or occupational therapist. In the brainstorming stage, participants provided 38 ideas in response to the focal prompt. The ideas were synthesised and structured into a final list of 45 unique statements. Six participants' sorting data were rejected due to either sorting less than 75% of statements or sorting inappropriately; for example, they sorted all statements into one pile, or the piles did not make sense due to unrelated statements being sorted together. Thirteen sorting data sets were approved for further analysis.

Due to participants logging in and out of the software for different activities, some participants skipped or did not get the chance to respond to all or any of the demographic questions. Of those that did, 11 participants identified as registered occupational therapists and indicated their clinical work experience in Trinidad and Tobago, which ranged from 2 to 16 years. Seventeen participants responded to the question about their current or intended area(s) of practice with answers including paediatrics (*n* = 13), stroke (*n* = 6), older adult care (*n* = 5), mental health (*n* = 4), and other (*n* = 3) which participants specified as orthopaedics, academia, and community development. Seventeen participants also identified whether they had experience with conducting research in occupational therapy, with 10 responding “yes” and 7 responding “no.”

### 3.2. Concept Map

**Figure 2:**
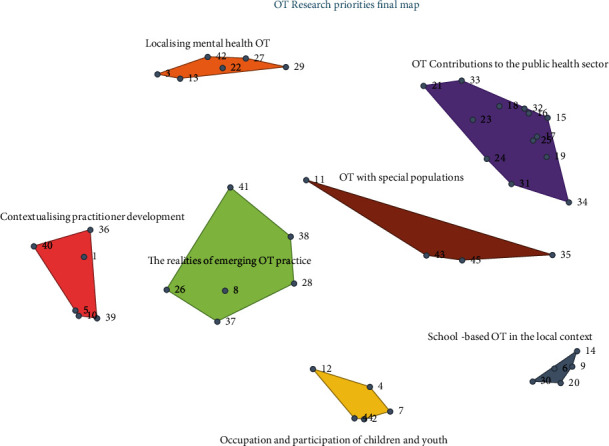
Point cluster map showing seven research priority themes. The map represents the distribution of each individual statement as numbered points.

Multidimensional scaling resulted in a point map with a stress value of 0.285, indicating goodness of fit of the sort data. Following hierarchical cluster analysis, several cluster maps were produced and reviewed by both the researchers and the gatekeeper. Consensus was reached on a 7-cluster solution with the following cluster names: Contextualising Practitioner Development, The Realities of Emerging OT Practice, Localising Mental Health OT, Occupation and Participation of Children and Youth, School-based OT in the Local Context, OT with Special Populations, and OT Contributions to the Public Health Sector. The point cluster map can be seen in Figure 2, where each numbered statement is represented by a point on the map. To ensure the resulting themes useable and workable in the context of occupational therapy in Trinidad and Tobago, cluster boundaries were redrawn such that statement #11 and statement #34 were included in the clusters which better represented those ideas [44].

### 3.3. Pattern Match

**Figure 3:**
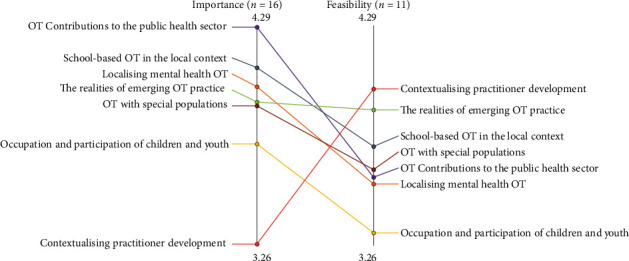
Pattern match comparing the mean importance and feasibility rating scores at cluster level. Clusters with higher mean scores are located closer to the top of the pattern match (*r* = −0.41).

A pattern match comparing the mean rating scores for importance and feasibility for each of the seven clusters can be seen in Figure 3. The clusters are arranged in ranked order along horizontal lines which represent importance (left) and feasibility (right). The pattern match shows that all clusters were rated higher for importance than feasibility, except for *Contextualising Practitioner Development* which was ranked as the least important priority area but the most feasible area to research. Differently, the cluster *OT Contributions to the Public Health Sector* was disparately ranked highest for importance but fifth out of seven for feasibility. *School-based OT in the Local Context* was considered the next highest priority area and was ranked third for feasibility. While *The Realities of Emerging OT Practice* was ranked fourth in importance but second for feasibility, comparatively these mean scores appear relatively close to each other on the pattern match.

### 3.4. Go-Zone Graphs and Statements

**Figure 4:**
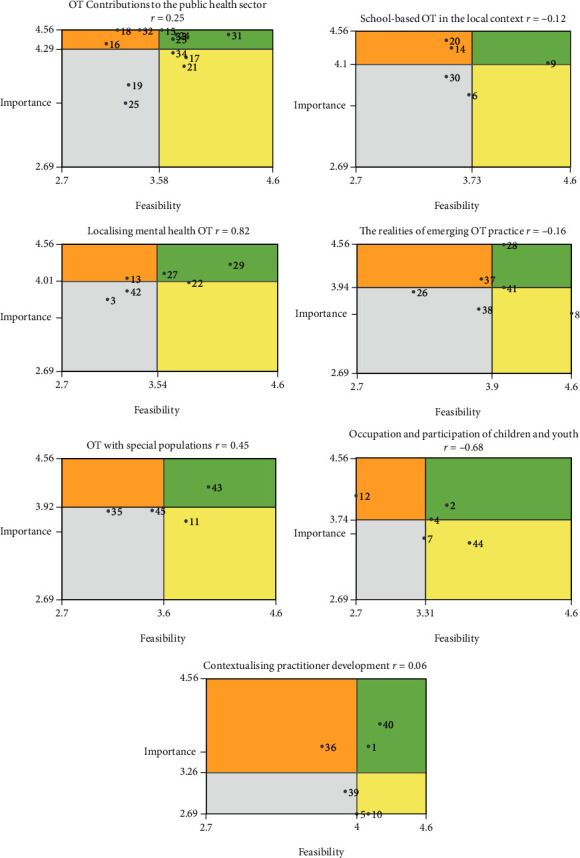
Go-zone plots for each of the seven clusters. Statements are represented as numbered points. The upper right quadrant of each plot, the “go-zone”, represents statements considered as research priorities while the upper left quadrant represents areas for future development. Importance (*n* = 16); feasibility (*n* = 11).

Figure 4 is a visual representation of the go-zone plots that were generated for each of the seven research priority clusters. Statements in the “go-zone” that were rated high importance and high feasibility were of interest for this study as they were considered priority research themes.  Table 1 presents this information in more detail showing a list of statements contained within each cluster and highlights those that were in “go-zones” as actionable priorities, as well as those that are high importance but slightly less feasible as areas for future development.

## 4. Discussion

This study engaged occupational therapy practitioners and students in a systematic and structured GCM project to identify occupational therapy research priorities within the local context of Trinidad and Tobago. The GCM process promoted shared research agenda setting by connecting ideas from various areas of practice, identifying ideas that were highly agreed upon, and adding value ratings to clusters and statements within. The participatory approach facilitated by the GroupWisdom™ software yielded results that represent local clinical and educational experiences which do not exist in the literature. The adoption of these research priorities by the occupational therapy profession in Trinidad and Tobago will determine how effective they are in promoting and advancing the profession through occupational therapy research.

While the sample size was small and differed across the stages of data collection, the findings are nevertheless representative of the occupational therapy population in Trinidad and Tobago, which is small and in early developmental stages [1]. Demographic data indicated that participants had a varying range of work experience and were therefore able to contribute research ideas from a broad range of perspectives. Although some participants left demographic questions unanswered, each participant was either a student or a therapist with local experience, since all participants were recruited as TTOTA members. Some clusters contained statements which were closely clustered together, indicating that they were frequently sorted together by participants. However, other clusters contained statements which represent broader conceptual themes, as demonstrated by more space between the points within it. Redrawing of cluster boundaries aided the production of seven distinct clusters. Perhaps a larger sample size and more participants taking part in the sorting stage of the study may have benefited the cluster map's understandability, negating the need to redraw cluster boundaries [44]. Nevertheless, the seven clusters indicate a common understanding among participants of the key areas for research development that reflect the current needs of the profession.

It can be said that all clusters and corresponding statements generated in this study are important and crucial to the growth of the evidence base due to the implications of occupational therapy being an emerging field in Trinidad and Tobago.  Table 1 illustrates this, as no statements in this study were rated lower than 2 (somewhat important) on the importance Likert scale. Rating the research ideas on importance alone would not have produced results that could be prioritised on actionability since participants thought all research ideas were important. Addition of the feasibility rating scores therefore produced more functional research priorities. The pattern match can act as a guide to future researchers, giving insight and comparison to the perceived importance and/or feasibility of specific research themes.

The researchers examined each cluster individually using data from the point cluster map, pattern match, go-zone graphs, and Table 1 to determine which research ideas should be prioritised. Future researchers are encouraged to use the pattern match to compare rating scores at cluster level and Table 1 to focus on a themed cluster, identify specific research topics, and consider its importance and feasibility rating. Although each themed cluster holds valuable information that can be explored in detail, for the purpose of this study, each cluster is discussed in terms of its general ability to be researched within the local context. The clusters are discussed in their ranked order of importance. While the feasibility rating scores for each cluster varied only between 3.3 and 4 out of 5, the rankings of the feasibility scores are discussed to help tease out the perceived feasibility for addressing the research priority themes for each of these clusters by the occupational therapy staff and students who took part.

### 4.1. OT Contributions to the Public Health Sector (13 Statements)

This cluster was rated the most important themed cluster and contained the largest number of statements. The relatively large portion of statements that fell within the go-zone for this cluster indicates that participants perceive several ideas within this cluster to be both important and feasible and therefore worthy of immediate research attention. These go-zone statements are all related to exploring the role, need, and benefits of occupational therapy in the public health sector in Trinidad and Tobago.

As of 2019, only three of the twenty-two practicing occupational therapists in the country worked within the public sector [1]. One of these therapists worked in a mental health hospital in Trinidad, and the other two worked in the acute hospital in Tobago covering mental health and physical disabilities. The remaining nineteen therapists worked within the private sector in Trinidad. Although the number of occupational therapy staff is expected to grow with new graduates entering the profession in 2020 and 2022 [1], there is a lack of public sector positions available to therapists which ultimately stifles growth of the profession [8]. A previous attempt has been made to integrate a paediatric occupational therapy clinic in a public sector hospital in Trinidad. However, this project came to a halt as governmental approval is still pending [8].

Despite the Trinidad and Tobago government's heavy investment of approximately 6% of its total GDP in healthcare, there are frequent reports on persistent shortages in staff, beds, and medicines within public sector hospitals [45]. These shortfalls have ensued a prosperous private healthcare sector which is not economically accessible to many people [45], resulting in an inequitable health environment [46].

The lack of occupational therapy representation in the public sector results in limited access to services and reinforces health inequities and occupational injustice. This might explain why participants agreed that this cluster was the most important research theme in this study. Research in this area may help spread awareness on the occupational therapy role and provide evidence on the potential benefits the profession may have on the public healthcare system. This evidence may warrant the allocation of public funds to expand occupational therapy services in the country.

This cluster was rated relatively low for feasibility in comparison to the other clusters, the fifth out of seven, which can be accounted for by the lack of staff practicing in this area. Perhaps with the implementation of more practitioners in the public healthcare sector over time, research would become more feasible to conduct. Although researchers could use international examples to evidence the importance of occupational therapy in public healthcare systems, it is crucial to highlight local perspectives to instigate governmental action. The United Kingdom Medical Research Council (MRC) provides a framework for developing complex interventions to improve health and healthcare which could be applied to developing, piloting, evaluating, and implementing occupational therapy services broadly in Trinidad and Tobago's healthcare system [47]. The original MRC guidance has been extended to include more guidance on intervention development [48]. Researchers interested in this area could use the MRC framework as a tool for action to guide the development and implementation of occupational therapy services in this setting.

### 4.2. School-Based OT in the Local Context (5 Statements)

This is the second most important themed cluster and the third most feasible, with only one out of its five statements appearing within the go-zone. This statement (#9 the correlation between accessing OT services and improved school performance) describes the relationship between children receiving occupational therapy and their school performance. Thirteen occupational therapists presently work in paediatrics in a private outpatient setting in Trinidad [1]. Due to majority of the workforce working with children, researching the impact of services on school performance is a relatively highly feasible project to undertake since this population already exists. Studying the impacts of occupational therapy services locally can help to inform practice and provide evidence when appealing to governmental ministries.

Statements within this cluster also relate to the implementation of occupational therapy within schools. As of 2019, only one occupational therapist worked in a public school in Trinidad [1]. This means that the Ministry of Education employs only one occupational therapist in the country. If parents outside of this school wanted to access occupational therapy for their children, they would have to do so through private clinics at great cost. This shortage in the education system may explain why this cluster was rated the second most important theme in the study. Integration into the public education system would likely require the Ministry of Education to offer jobs within schools and provide schools with the resources required to facilitate therapy.

The need for school-based research that exists within the political, cultural, and structural context of a country is emphasised in the literature [49, 50]. The development of school-based occupational therapy is also strongly influenced by the way services are provided and the educational structures, policies, and systems in place [49, 50]. Future researchers must therefore consider the local context fully.

### 4.3. Localising Mental Health OT (6 Statements)

This cluster was rated the third most important and the second to last feasible research theme of all statements linked to occupational therapy in various mental health settings. As mentioned previously, there are two occupational therapists in Tobago covering mental health and only one in Trinidad within a public-sector hospital, the only specialised mental health hospital in the nation [1]. In Trinidad and Tobago, there are very few mental health practitioners with services limited to out-of-date institutionalised care mostly provided by the MoH [51]. The general and other county hospitals accommodate small inpatient psychiatric units for acute mentally ill persons. Older people with chronic mental illness have access to four extended care centres which can be understaffed with personnel who may not have been trained in mental health care [51]. Some additional outpatient mental health services are offered across the country [52]. Comparable to the two previous clusters, the scarce community of mental health occupational therapists may explain why participants believed this to be a relatively high important area for future research. Although this cluster was rated relatively low for feasibility, further examination of the statements (Figure 4 and Table 1) shows that go-zone statements are feasible to study and could be a good starting point for interested researchers. These are statement #29 (The role and need for OT in mental health settings in Trinidad and Tobago) and statement #27 (The role of OT in promoting independence with ADL's and IADL's in mental health institutions to contribute to reintegration into society).

### 4.4. The Realities of Emerging OT Practice (6 Statements)

All statements in this cluster related to occupational therapy services more generally in Trinidad and Tobago, compared to other clusters concerned with specific areas of practice. This cluster was rated fourth in importance and second for feasibility. The two statements in the go-zone were rated highly feasible and can be considered for immediate research action. Specifically, statement #28 (Accessibility of OT services by members of the general public in Trinidad and Tobago) was among the most important research ideas across all statements in this study and should be considered for immediate response (Table 1). Additionally, despite not being included in the go-zone due to a relatively lower importance score, statement #8 (What is OT? A qualitative exploration of the public's knowledge and awareness of OT services available in Trinidad and Tobago) was rated as the most feasible statement overall in this study and is therefore strongly recommended as a priority for investigation. Statement #8 pertains to public awareness of occupational therapy services and can be conducted with the already existing population. This would not require further development of services and is actionable now. Perhaps, statements #28 and #8 are activities that can be led by the professional association as they pertain to issues of general accessibility and awareness of the profession overall. In addition, statement #26 (Balancing the small ratio of OTs to client: searching for an effective mode of service delivery in Trinidad and Tobago) relates to service delivery in the country and may be more appropriately researched by the professional body. It can be argued that all occupational therapists in Trinidad and Tobago are in role emerging positions. Enhancing the profession within the local context through awareness building, development, and advancement of the profession through research and practice is mandated by the Code of Ethics [6]. Researching this cluster could help to build a picture of where the profession is at in its development.

### 4.5. OT with Special Populations (4 Statements)

This is the smallest cluster containing four statements, each related to different populations' needs relevant to occupational therapy. The distance between this cluster's statements is shown on the point cluster map (Figure 2), which indicates that participants did not sort these statements together as frequently as they did other statements, possibly because the ideas were not perceived as related in comparison. Researchers interested in the specific populations in this cluster should further analyse the feasibility of research explicitly for their chosen topic. Statement #43 (The role of OT in navigating accessibility for persons with physical disabilities), within the go-zone, is an actionable research priority that can have lasting effects for many persons with disabilities and may be a useful place to begin.

### 4.6. Occupation and Participation of Children and Youth (5 Statements)

The statements within this cluster are all related to the impact of external influences on young people's ability to engage and participate in occupations. This cluster was rated second to last for importance and lowest for feasibility. These relatively low ratings may be due to the difficulty of studying these external influencers as they may not be currently in the immediate scope of occupational therapy in Trinidad and Tobago. While the feasibility score is the lowest out of all the clusters (3.3/5), the feasibility scores overall did not vary much between clusters. The statements within the go-zone can be feasibly studied since the majority of occupational therapists in the country work in paediatrics and have access to this demographic. There are approximately 3,302 children with disabilities in Trinidad and Tobago (Ministry of People and Social Development as cited in [8]). Of these children, 99% go untreated for occupational therapy due to the scarcity of services [8]. Despite the larger proportion of practitioners working in paediatrics, the lack of services within this population persists. Research into this cluster has the potential to impact the lives of many children and their families, as well as support and evidence the field of occupational therapy.

### 4.7. Contextualising Practitioner Development (6 Statements)

This cluster was rated the least important but the most feasible research theme in this study. The comparably lower importance score may be because the other clusters in this study were considered more impactful on the immediate growth of the field. The comparably high feasibility score may be because the research sample is easily accessible due to the willingness and requirement of practitioners to participate in research [6]. The statements in the go-zone are a recommended starting point for research within this cluster and pertain to professional development issues. Practitioner development in Trinidad and Tobago is aided by the TTOTA who actively arrange education and training opportunities, provide research and resources, and support new clinics [53]. Researchers could potentially liaise with the TTOTA for guidance and support in this research area. Furthermore, it may be in the interest of the local professional body to undertake some of the research ideas proposed in this cluster, such as statement #36 (Mapping the CPD opportunities for OT according to current, prevalent health and social conditions requiring OT input).

Three of the five statements in this cluster are concerned with native practitioners working outside the country. While these statements were not rated highly for importance, they were highly feasible and should be considered for future research. One in five persons born in Trinidad and Tobago emigrate contributing to brain drain of the country [54, 55]. From these research statements, it can be said that brain drain affects occupational therapy and is an important consideration for those involved in development of the profession locally.

### 4.8. Strengths and Limitations

The biggest strength of this study is its contribution to knowledge concerning research and development of occupational therapy locally in Trinidad and Tobago. This study sets the stage for future research in a country that has had such a limited evidence base of its own. It provides a guideline for future researchers and can provide research topics for master's students, who, being so new to the field, may have a limited understanding of the priority ideas. The seven identified research priority themes portray the current needs of the profession and have the potential for lasting effects on its growth. It should be recognised that with growth, it is expected that research priorities could change. The GCM method strengthens the representation of participant perspectives through its participatory approach, giving voice to a diverse set of experiences. This study, however, is limited by the many stages of data collection and the time required to participate. Some participants did not complete all stages, including the demographic questions, resulting in some missing data. In addition, there were relatively low numbers of participants in this study compared with other GCM studies [32]. This may have influenced the results in several ways. Firstly, the brainstorming stage finished after all participants had taken part. More participants may have yielded a greater number of ideas. We, therefore, cannot be certain that data saturation was achieved. Second, there was little variation in the overall rating scores for each cluster, in particular feasibility. It is possible that a greater number of participants may have produced greater variation in the overall cluster rating scores.

## 5. Conclusions

This study used a systematic group concept mapping approach asking occupational therapy practitioners and students in Trinidad and Tobago to identify research ideas in their field. The findings highlight seven research priority themes for the occupational therapy profession in Trinidad and Tobago: Contextualising Practitioner Development, The Realities of Emerging OT Practice, Localising Mental Health OT, Occupation and Participation of Children and Youth, School-based OT in the Local Context, OT with Special Populations, and OT Contributions to the Public Health Sector. Research ideas rated highly on importance and feasibility have been identified in go-zones and are suggested starting points for immediate research. It is hoped that future researchers will use the findings generated in this study to decide on meaningful research topics that will expand the occupational therapy evidence base in the country. Future research is required to assess the effectiveness of these research priorities on the growth and development of the local profession.

## Figures and Tables

**Table 1:** Mean importance and feasibility rating scores for clusters and statements.

	Statements and clusters	Importance (1-5)	Feasibility (1-5)
	**OT contributions to the public health sector**	**4.29**	**3.58**
15^a^	Benefits of rehabilitation teams in the public health sector in improving health outcomes	4.53	3.67
18^b^	The impact of OT services in public sector hospitals on minimizing long-term costs	4.53	3.42
32^b^	The benefits of inpatient OT services in preventing repeated hospital visits and declining health and wellness	4.53	3.50
24^a^	Benefits of promoting OT in the public hospitals in Trinidad and Tobago	4.47	3.77
31^a^	The role and need for OTs in multidisciplinary teams	4.47	4.25
23^a^	The impact of OT on the transition from hospital care to home to help minimize the chance of readmittance	4.41	3.67
33^a^	OT in outpatient settings: how services can contribute to the prevention of hospital readmission and declining health	4.41	3.75
16^b^	Reducing readmission rates: the impact of rehabilitation teams in the public health sector	4.35	3.25
34	The role of community OT in preventing deteriorating health and recurring hospital visits	4.24	3.75
17	Examining whether rehabilitation teams in the public health sector contribute to improving quality of life	4.18	3.85
21	How can OTs contribute to public outpatient clinics in Trinidad and Tobago?	4.06	3.83
19	An exploration of the benefits of OT in various public sector roles	3.82	3.33
25	Linking hand rehabilitation and OT to clients after a stroke within public hospitals	3.59	3.31

	**School-based OT in the local context**	**4.10**	**3.73**
20^b^	The benefits of OT in public schools	4.41	3.58
14^b^	OT in schools: a system that matches our unique population needs and the number of OTs we currently have	4.31	3.62
9^a^	The correlation between accessing OT services and improved school performance	4.12	4.33
30	The impact of sensory integration therapy on school performance of children with autism spectrum disorders	3.94	3.50
6	The adherence to OT recommendations by schools and teachers for children ages 3-7	3.71	3.75

	**Localising mental health OT**	**4.01**	**3.55**
29^a^	The role and need for OT in mental health settings in Trinidad and Tobago	4.25	4.23
27^a^	The role of OT in promoting independence with ADL's and IADL's in mental health institutions to contribute to reintegration into society	4.13	3.58
13^b^	The effect of mental health OT in low income youth and adolescents	4.06	3.31
22	Exploring the benefits of OT in mental health within the public sector	4.00	3.85
42	The impact of OT interventions in mental health settings: from acute to outpatient	3.88	3.31
3	The use of OT theoretical models of practice in mental health settings in Trinidad and Tobago	3.76	3.25

	**The realities of emerging OT practice**	**3.94**	**3.90**
28^a^	Accessibility of OT services by members of the general public in Trinidad and Tobago	4.53	4.00
37^b^	An overview of OT in Trinidad and Tobago: barriers and facilitators for access to OT services by health conditions and service user demographics	4.06	3.92
41^a^	The link between OT-conducted staff training and the well-being and function of older adults in nursing homes	3.94	4.00
26	Balancing the small ratio of OTs to client: searching for an effective mode of service delivery in Trinidad and Tobago	3.88	3.33
38	What are the functional outcomes of OT services across health conditions and service user demographics receiving OT input?	3.65	3.82
8	What is OT? A qualitative exploration of the public's knowledge and awareness of OT services available in Trinidad and Tobago	3.59	4.58

	**OT with special populations**	**3.92**	**3.62**
43^a^	The role of OT in navigating accessibility for persons with physical disabilities	4.18	4.00
45	The importance of the OT role in conducting wheelchair assessments for varying health conditions	3.88	3.50
35	OT intervention straight from the Neonatal Intensive Care Unit: the relationship between immediate access to OT and premature babies' needs for services later on	3.88	3.18
11	The impact of OT interventions on people with functional neurological disorders in Trinidad and Tobago	3.75	3.83

	**Occupation and participation of children and youth**	**3.74**	**3.31**
12^b^	OT and crime prevention in “at-risk” youth	4.06	2.75
2^a^	The relationships between the frequency of accessing outdoor and sport activities and the physical development of children ages 6-10 in Trinidad and Tobago	3.94	3.50
4^a^	The impact of family income on opportunities for children and adolescents to access leisure occupations in Trinidad and Tobago	3.76	3.38
7	The impact of stigma on accessing therapeutic interventions by parents of children ages 3-7 who have been recommended OT	3.53	3.33
44	The use of aqua therapy by OTs in children with cerebral palsy	3.47	3.67

	**Contextualising practitioner development**	**3.26**	**4.00**
40^a^	OT practitioner burn out: causes and strategies for prevention	3.94	4.17
1^a^	“Life long learner”: what are the CPD opportunities for OT practitioners in Trinidad and Tobago?	3.65	4.17
36^b^	Mapping the CPD opportunities for OT according to current, prevalent health, and social conditions requiring OT input	3.65	3.75
39	“National OTs practising overseas”: prevalence, areas of practice abroad and potential areas for local involvement back in Trinidad and Tobago	3.06	3.92
5	“I went away to come back”: the experience of Trinidadian OTs who studied away and now practice in Trinidad and Tobago	2.76	4.00
10	“Home or away” an exploration with Trinidadian OT graduates: what influences the choice to practice at home or abroad?	2.76	4.08

The numbers in the left column coincide with the statement numbers depicted in the point cluster map (Figure 2) and the go-zone plots (Figure 4). ^a^Prioritised statement rated above the mean for both importance and feasibility. ^b^Area for future development rate high importance but deemed less feasible. Cluster names and their corresponding importance and feasibility scores are in bold.

## Data Availability

The data used to support the findings of this study are available from the corresponding author upon request.
